# Class III Alcohol Dehydrogenase Modulates Renal Parietal Epithelial Cell Transformation During Chronic Alcohol Consumption in Mice

**DOI:** 10.3390/ijms26136279

**Published:** 2025-06-29

**Authors:** Midori Katsuyama, Takahisa Okuda, Masamichi Ishizaki, Kentaro Wada, Motoyo Maruyama, Toshio Akimoto, Youkichi Ohno, Takahito Hayashi, Takeshi Haseba

**Affiliations:** 1 Department of Legal Medicine, Graduate School of Medical and Dental Sciences, Kagoshima University, 8-35-1 Sakuragaoka, Kagoshima 890-8544, Japan; 2 Department of Legal Medicine, Nihon University School of Medicine, 30-1 Oyaguchi-Kamicho, Itabashi-ku, Tokyo 173-8610, Japan; 3 Department of Plastic, Reconstructive & Aesthetic Surgery, Nippon Medical School, 1-1-5 Sendagi, Bunkyo-ku, Tokyo 113-8602, Japan; 4 Department of Analytic Human Pathology, Nippon Medical School, 1-1-5 Sendagi, Bunkyo-ku, Tokyo 113-8602, Japan; 5 Division of Nephrology and Dialysis, Department of Internal Medicine, Nippon Kokan Fukuyama Hospital, 1844 Tsunoshita, Daimon-cho, Fukuyama, Hiroshima 721-0927, Japan; 6 Division of Laboratory Animal Science, Nippon Medical School, 1-1-5 Sendagi, Bunkyo-ku, Tokyo 113-8602, Japan; 7 Department of Legal Medicine, Nippon Medical School, 1-1-5 Sendagi, Bunkyo-ku, Tokyo 113-8602, Japan; 8 Department of Legal Medicine, Kanagawa Dental University, 82 Inaoka-cho, Yokosuka, Kanagawa 238-8580, Japan

**Keywords:** alcohol, alcohol dehydrogenase, ADH3, kidney, renal pathological abnormality

## Abstract

Class III alcohol dehydrogenase (ADH3), primarily localized in the liver and kidney, contributes to alcohol metabolism during chronic alcohol consumption (CAC). However, its role in kidney function remains unclear. This study investigated renal morphological changes associated with ADH3-mediated alcohol metabolism. Nine-week-old male wild-type (WT) and ADH3-deficient (*Adh3^-/-^*) mice were administered 10% ethanol for 1 month. Histological analyses were performed using periodic acid–Schiff (PAS) staining and electron microscopy. Serum biochemical parameters were also assessed. In WT mice, CAC induced an increase in cuboidal parietal epithelial cells (PECs) in Bowman’s capsule, along with elevated testosterone levels in both serum and urine. *Adh3^-/-^* mice showed increased PECs even in the control group, with similarly elevated serum testosterone in both control and ethanol-treated groups. These findings suggest that ADH3 is involved in testosterone metabolism, and that that metabolism is suppressed by CAC because ADH3 shifts toward ethanol metabolism. The resulting testosterone elevation may contribute to PEC proliferation. An increase in PECs observed even in *Adh3^-/-^* control mice may also be caused by the lack of testosterone metabolism via ADH3. Thus, renal ADH3 may protect kidney structure through testosterone metabolism, but its role may be disturbed by CAC. This study highlights the role of ADH3 in the relationship between physiological steroid metabolism and alcoholic pathological abnormality in the kidney.

## 1. Introduction

The nephron is the microscopic structural and functional unit of the kidney, consisting of the renal corpuscle, proximal convoluted tubule, loop of Henle, and distal convoluted tubule. The renal corpuscle, where plasma filtration occurs, is composed of two main structures: the glomerulus and Bowman’s capsule. The parietal epithelial cells (PECs) lining Bowman’s capsule form a layer of flat epithelial cells that are continuous with the proximal tubular epithelium. PECs can undergo morphological changes, swelling and transforming into cuboidal-shaped cells with a crescent formation. This phenomenon has been specifically observed in sexually mature male laboratory mice [1,2,3]. These phenomena are infrequently observed in female mice. This observation suggests that the cuboidal transformation of PECs in male mice is associated with increased testosterone secretion during sexual maturation. Such changes are considered to represent a form of physiological adaptation. Moreover, these cuboidal PECs resemble the epithelium of the proximal convoluted tubule [4]. Although cuboidal PECs resemble proximal tubule cells, the significance of their reabsorptive function within the glomerulus—which primarily serves as a filtration unit—remains unclear. Furthermore, it is still uncertain whether these cells are indeed proximal tubules or a distinct cell population. Conversely, other studies suggest that the appearance of cuboidal PECs may be related to pathological conditions such as glomerulosclerosis [5]. Therefore, whether the presence of cuboidal PECs reflects a physiological adaptation or a pathological change remains unclear and warrants further investigation.

In general, alcohol consumption increases testosterone levels in vivo, whereas Mårdh et al. reported that testosterone allosterically regulates alcohol metabolism via alcohol dehydrogenases (ADHs; EC 1.1.1.1) [6]. Among the six ADH classes, class I (ADH1) is the primary enzyme responsible for alcohol metabolism. However, class III ADH (ADH3), which has the highest K_m_ for ethanol among the ADH family, also contributes to systemic alcohol metabolism in a dose-dependent manner in vivo [7,8], particularly during chronic alcohol consumption (CAC) [8,9]. ADH3 is expressed at the highest levels in the liver, with the second-highest expression observed in the kidney [10]. Recent studies have shown that low blood ethanol concentrations can upregulate ADH protein expression and activity in the kidney [8,11], suggesting that ADH3 may also participate in renal alcohol metabolism. Our previous work demonstrated that ADH3 exacerbates kidney damage during alcohol metabolism [12]. Notably, Swank et al. reported that androgens increase ADH activity in the kidney [13]. On the basis of these observations, we hypothesize that ADH3 increases testosterone levels through alcohol metabolism, thereby promoting the cuboidal transformation of PECs. Furthermore, evaluating whether this phenotypic change in PECs represents a physiological adaptation or a pathological alteration is of significant relevance in the field of alcohol-related metabolism research and renal pathology. The cuboidal transformation of PECs is occasionally observed not only in mice but also in humans, suggesting that the present study may also provide insights into the mechanisms underlying PECs cuboidalization in humans.

Based on these findings, we examined the relationship between androgens and the transformation of flat PECs into cuboidal PECs in Bowman’s capsule, particularly in the context of growth and alcohol exposure. To date, no studies have investigated the specific effects of alcohol consumption and ADH activity on PEC morphology in the kidney. Therefore, we conducted an experimental study using ethanol-administered mice with different ADH genotypes—wild-type (WT) and ADH3-deficient (*Adh3^-/-^*) C57BL/6N mice—to investigate whether ADH3-mediated alcohol metabolism influences the transformation of flat PECs into cuboidal PECs within the renal glomeruli.

## 2. Results

### 2.1. Alcohol Induces an Increase in Bowman’s Capsules with Cuboidal Parietal Epithelial Cells

**Figure 1:**
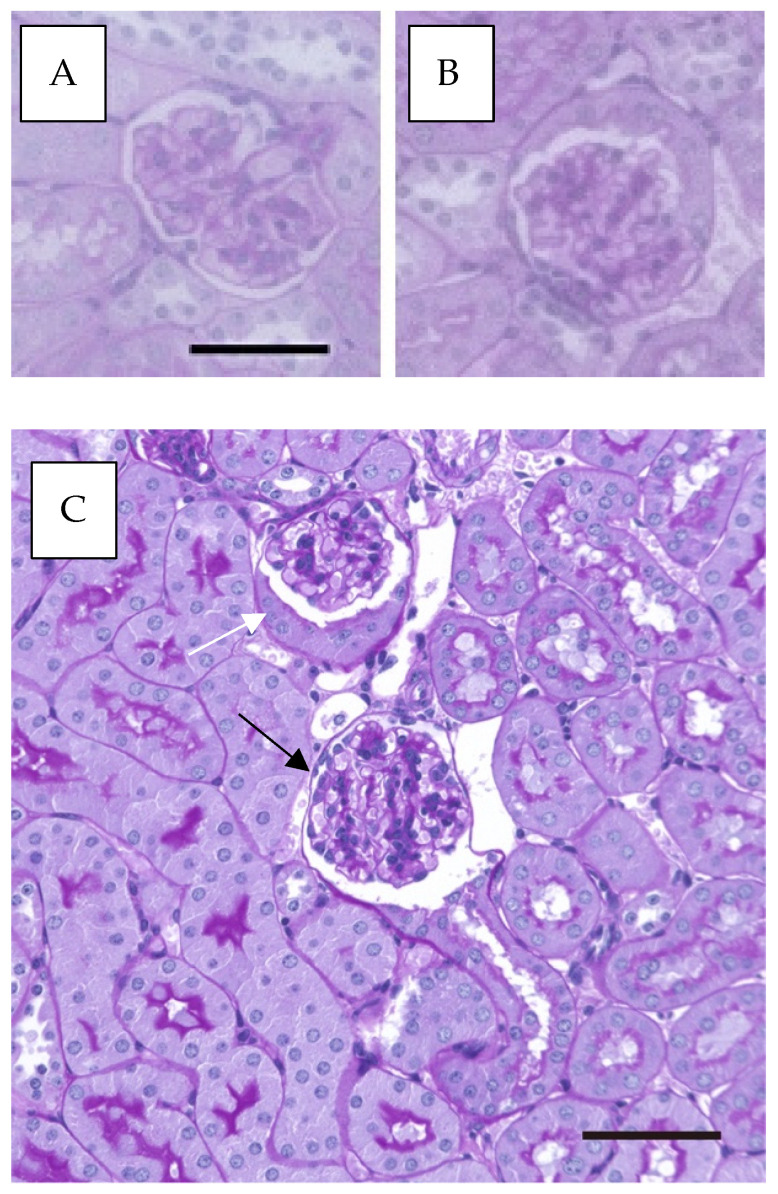
Increase in Bowman’s capsule with cuboidal parietal epithelial cells (PECs) of WT mice, with or without alcohol exposure. (**A**) Bowman’s capsule with flat PECs, stained with periodic acid–Schiff (PAS). (**B**) Bowman’s capsule with cuboidal PECs, stained with PAS. (**C**) Bowman’s capsule showing both flat and cuboidal PECs, stained with PAS. Black arrow in (**C**) shows Bowman’s capsule with flat PECs. White arrow in (**C**) shows Bowman’s capsule with cuboidal PECs. The black scale bars in (**A**,**C**) represents 50 µm.

Parietal epithelial cells (PECs) in Bowman’s capsule can undergo cellular activation, characterized by enhanced proliferative, migratory, and extracellular matrix-producing capacities [5]. In this study, we observed morphological changes in PECs following alcohol consumption in WT mice. As shown in Figure 1, PECs lining Bowman’s capsule were typically flat (Figure 1A, and black arrow in Figure 1C); however, in alcohol-treated mice, a transformation to cuboidal PECs was observed (Figure 1B and white arrow in Figure 1C).

**Figure 2:**
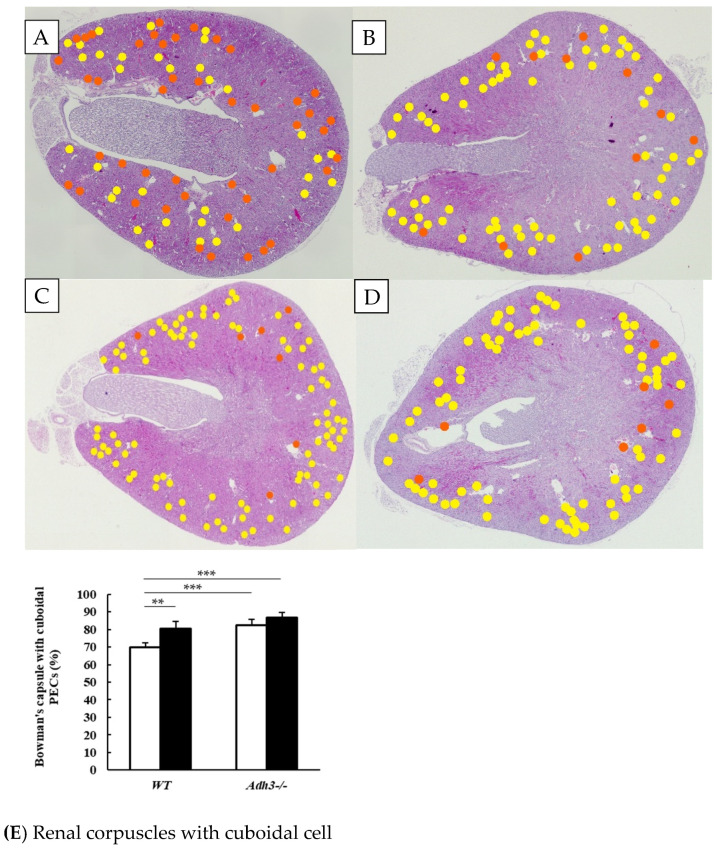
Number of Bowman’s capsules with flat and cuboidal PECs (**A**–**D**); The WT mice administered water shown in (**A**); WT mice administered ethanol shown in (**B**); *Adh3^-/-^* mice administered water shown in (**C**); *Adh3^-/-^* mice administered ethanol shown in (**D**). In panels (**A**–**D**), orange circles indicate Bowman’s capsules with flat PECs, and yellow circles indicate Bowman’s capsules with cuboidal PECs. The Bowman’s capsule with cuboidal PECs observed by hematoxylin and eosin (HE)-staining. (**E**) Ratio of Bowman’s capsules with cuboidal PECs to glomerular PECs. White bars represent WT (C) and *Adh3^-/-^* (C) as the control group; black bars represent WT (E) and *Adh3^-/-^* (E) as the alcohol group. Values represent means ± SD; n = 5 mice per group. ** *p* < 0.01, and *** *p* < 0.001 between control (C) and alcohol (E) groups, as assessed by a one-way analysis of variance with Tukey’s honest significant difference as a post hoc test. P-values are shown for comparisons between groups WT (C), WT (E), *Adh3^-/-^* (C), and *Adh3^-/-^* (E).

Among the control groups, the number of Bowman’s capsules with cuboidal PECs was significantly higher in *Adh3^-/-^* (C) mice compared to WT (C) mice (Control group: WT (C) and *Adh3^-/-^* (C)). In WT mice, CAC significantly increased the number of Bowman’s capsules with cuboidal PECs (Figure 2A,B,E). However, in *Adh3^-/-^* mice, alcohol consumption did not significantly alter this number (Figure 2C–E; CAC group: WT (E) and *Adh3^-/-^* (E)).

### 2.2. Bowman’s Capsule with Cuboidal Parietal Epithelial Cells Observed by Electron Microscopy

**Figure 3:**
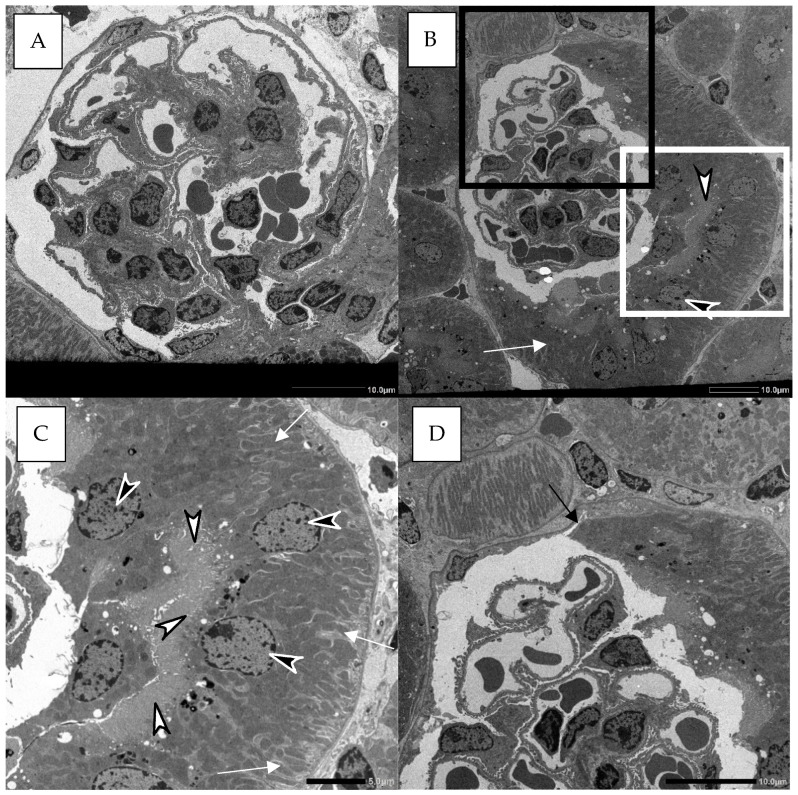
Electron microscopy images of Bowman’s capsules with cuboidal PECs in control (**A**) and ethanol-treated WT mice (**B**–**D**). The Bowman’s capsule with cuboidal PECs observed by electron microscopy. The white box in (**B**) is enlarged in (**C**). The black box in (**B**) is enlarged in (**D**). White arrows in (**B**,**C**) indicate mitochondria-rich cuboidal PECs. White arrowheads in (**B**,**C**) highlight microvilli formation in the cuboidal PECs. Black arrowheads in (**B**,**C**) show nuclei aligned around the microvilli. (**D**) The black arrow indicates the origin of cuboidal PECs within flat PECs. The black scale bar represents 10 µm in (**A**,**B**,**D**) and 5 µm in (**C**), respectively.

Bowman’s capsules with PECs were examined using an electron microscope (Figure 3). In control WT mice, PECs were predominantly flat (Figure 3A), whereas in ethanol-treated mice, they appeared cuboidal in shape (Figure 3B,D). These cuboidal PECs closely resembled proximal tubular epithelial cells, characterized by abundant mitochondria and the presence of a brush border. Notably, the nuclei were aligned along the brush border (Figure 3B,C). Additionally, the origin of the cuboidal PECs, similar to those in the proximal tubule, extended inward from regions initially occupied by flat PECs (Figure 3D).

### 2.3. Alcohol Alters Testosterone Parameter

**Figure 4:**
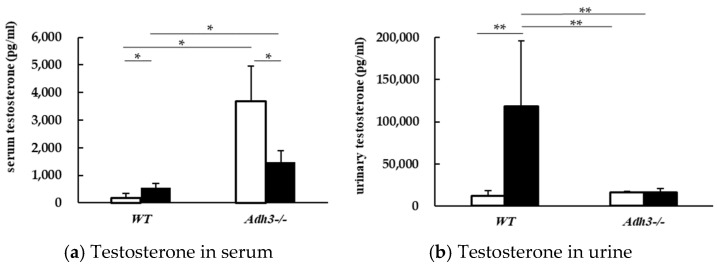
(**a**) Serum testosterone levels in WT and *Adh3^-/-^* mice. (**b**) Urinary testosterone levels in WT and *Adh3^-/-^* mice. White bars represent WT (C) and *Adh3^-/-^* (C): control group; black bars represent WT (E) and *Adh3^-/-^* (E): alcohol group. Values represent means ± SD; n = 5 mice per group. * *p* < 0.05, ** *p* < 0.01 between control (C) and alcohol (E) groups, as assessed by one-way analysis of variance with Tukey’s honest significant difference as a post hoc test. *P*-values are shown for comparisons between groups: WT (C), WT (E), *Adh3^-/-^* (C), and *Adh3^-/-^* (E).

Regarding hormonal changes, serum and urinary testosterone levels were significantly increased in WT mice after alcohol consumption (Figure 4). Conversely, serum testosterone levels were significantly decreased in *Adh3^-/-^* mice after alcohol exposure (Figure 4a). Notably, *Adh3^-/-^* mice showed significantly higher serum testosterone levels compared to WT mice under control conditions (Figure 4b).

## 3. Discussion

Previous studies have demonstrated that flat PECs in the murine Bowman’s capsule can transform into proximal tubule-like cuboidal PECs [5]. This transformation has been reported to occur more frequently in male mice than in females [10], likely due to higher testosterone levels [4]. Elevated testosterone has been associated with the cuboidal transformation of PECs. In this study, electron microscopy revealed an increase in cuboidal PECs and brush borders in Bowman’s capsule following alcohol consumption. Additionally, serum and urinary testosterone levels were considerably higher in WT (E) mice compared to WT(C) mice. These findings suggest that the alcohol-induced elevation of testosterone levels may contribute to the increase in cuboidal PECs.

The role of ADH3 in the kidney has been underexplored, with few studies addressing its impact. Katsuyama et al. demonstrated that ADH3 may contribute to renal pathological changes, including increased glomerular permeability induced by CAC, potentially exacerbating alcoholic kidney disorders [12]. Dinu et al. showed that ethanol consumption (2 g/kg) increased ADH activity in the kidney [14]. This study investigated the renal morphological features in ADH3-deficient mice with or without alcohol consumption. We observed a significant increase in the number of cuboidal PECs in the Bowman’s capsules of *Adh3^-/-^* (C) mice, and their serum testosterone levels were significantly increased compared to WT (C) mice. These findings suggest that ADH3 regulates testosterone levels, potentially via its role in alcohol metabolism. It is possible that alcohol increases testosterone levels though ADH3-dependent metabolism, leading to the cuboidal transformation of PECs. Previous studies have indicated that testosterone allosterically regulates ethanol oxidation by alcohol dehydrogenase containing the γ subunit [6]. Furthermore, 19-hydroxytesosterone is a competitive substrate for ethanol in ADH [15]. In testosterone metabolism, the NAD-dependent oxidation of 19-hydroxytestosterone to 19-oxotestosterone is mediated by ADH; therefore, it may be competitively inhibited by ethanol, which is also NAD-dependently oxidized by ADH. Thus, CAC is thought to increase concentrations of both 19-hydroxytestosterone and testosterone.

**Figure 5:**
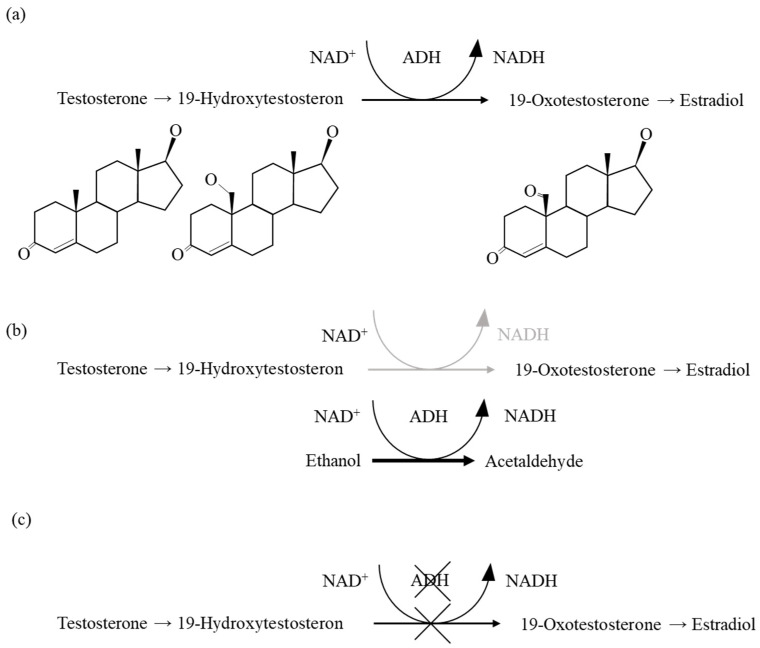
Schematic pathway of cuboidal parietal epithelial cell (PEC) formation caused by alcohol or ADH3 deficiency. (**a**) Physiological testosterone metabolism pathway. ADH functions as an NAD-dependent oxidase, converting 19-hydroxytestosterone to 19-oxotestosterone [15]. (**b**) During CAC, ethanol acts as a competitive substrate for ADH in testosterone metabolism, inhibiting the oxidation of 19-hydroxytestosterone. This suppression of testosterone metabolism leads to increased levels of 19-hydroxytestosterone and testosterone, which in turn promote the formation of cuboidal PECs. (**c**) In the absence of ADH3, testosterone metabolism is impaired, resulting in the accumulation of 19-hydroxytestosterone and testosterone, which also contributes to the formation of cuboidal PECs.

This study indicated that ethanol consumption in WT (E) mice significantly increased serum testosterone levels compared to WT (C) mice. However, in *Adh3^-/-^* (C) mice, the lack of ADH3 hindered testosterone metabolism, resulting in a significantly higher serum testosterone level. Based on our findings, we propose a two-step mechanism by which testosterone affects the mouse renal system (Figure 5). Consequently, the high testosterone levels observed in the *Adh3^-/-^* (C) mice were associated with an increased number of cuboidal PECs.

Recent reports have proposed two mechanisms for the formation of cuboidal PECs: physiological differentiation [4,10] and renal dysfunction [5,16,17]. Hanker et al. suggested that an increase in PECs, associated with foot process effacement and glomerulosclerosis, could result in renal dysfunction [5,16,17]. However, despite the increased cuboidal PECs in the *Adh3^-/-^* (C) mice, they did not exhibit signs of renal dysfunction [12]. Additionally, while the *Adh3^-/-^* (E) mice showed some mitochondrial collapse in proximal tubular cells under electron microscopy, the damage was less severe compared to the WT (E) mice. This suggests that alcohol consumption has a less significant impact in the absence of kidney ADH3.

Previous studies have reported that gross structural abnormalities or microscopic lesions in the kidney were not evident during alcohol consumption [18], suggesting that renal dysfunction may be less apparent. However, alcohol consumption could lead to albumin and proteins leakage into the urine, as well as increased blood urea nitrogen (BUN) and serum creatinine levels [19]. In our previous study, albumin and protein were significantly leaked into the urine after alcohol consumption in WT mice [12], consistent with these findings. In contrast, ADH3 deficiency did not significantly lead to urinary albumin and protein leakage. Since electrolyte balance and osmotic pressure were generally stable, it is likely that the reabsorptive function of the proximal tubule remained normal in ADH3-deficient mice. Therefore, alcohol consumption causes urinary abnormalities rather than overt renal disorders in this experimental condition. Accordingly, WT mice with alcohol consumption exhibited more abnormal renal function parameters compared to *Adh3^-/-^* (E) mice. Therefore, our findings suggest that ADH3 metabolizes alcohol during CAC and may exacerbate renal dysfunction.

Furthermore, alcohol consumption may induce an increased number of cuboidal PECs in the presence of ADH3. Notably, in the absence of ADH3 and without alcohol ingestion, high serum testosterone levels were also associated with an increase in cuboidal PECs. During alcohol metabolism, ADH3 in the kidney may be involved in the metabolism of ethanol, and its primary physiological role in testosterone metabolism could be inhibited. This inhibition, in turn, may contribute to pathological abnormalities in the kidney. Therefore, ADH3 may be a key factor in alcohol-induced renal dysfunction. Although it has been previously reported that alcohol consumption increases testosterone levels, our study is the first to identify a role for ADH3 in this process. Furthermore, the finding that its mechanism may induce morphological changes in PECs provides important new insights from the perspective of renal pathology.

ADH3 induces pathological changes in the renal proximal tubules by metabolizing alcohol at this site, leading to increased glomerular permeability during CAC. However, in *Adh3^-/-^* mice, no significant changes were observed in podocyte dilation, creatinine levels, or BUN, indicating the absence of overt renal dysfunction [12].

For example, it takes time for diabetic nephropathy to become clinically recognizable as renal damage. To better compare our findings, we plan to administer alcohol for one year instead of one month in future studies. Additionally, since short-term and long-term alcohol consumption may have different effects on testosterone levels, a one-year alcohol administration study may provide further insight into the long-term impact of alcohol and ADH3 on testosterone metabolism and renal morphology.

## 4. Materials and Methods

### 4.1. Animals

An ADH3-deficient mouse (*Adh3^-/-^*) contained homozygous null mutants for the *Adh3* genes. This mouse was originally obtained from the Burnham Institute, USA, in 1999 [20], and subsequently backcrossed with the C57BL/6N background strain (WT mice, Sankyo Lab., Co., Ltd., Tokyo, Japan) for up to 13 generations. The congenic *Adh3^-/-^* strain has been maintained at Nippon Medical School (Tokyo, Japan) since 2013 and has been bred at Kagoshima University (Kagoshima, Japan) since 2020.

Mice of two genotypes (WT and *Adh3^-/-^*) were weaned at 4 weeks of age and housed in groups of 4–6 per cage under specific pathogen-free conditions. The environment was maintained at 24 °C ± 2 °C with a 14 h light/10 h dark cycle starting at 7:00 AM. Mice were provided a standard rodent diet (MF pellets, Oriental Yeast Co., Ltd., Tokyo, Japan) containing 12.5% fat calories and water ad libitum. For each experiment, five mice were used per group.

All animal experiments were conducted in compliance with the ARRIVE guidelines and were approved by the Institutional Committee of Laboratory Animals at Nippon Medical School (Approval No. 28-001) and the Animal Ethics Committee of the Division of Laboratory Animal Resources and Research at Kagoshima University (Approval No. MD23061) following the National Institutes of Health Guide for the Care and Use of Laboratory Animals (NIH Publication No. 8023, revised 1978).

### 4.2. Chronic Alcohol Consumption Experiment

CAC experiments were performed by providing 9-week-old male mice with a 10% (*w*/*v*) ethanol solution in water ad libitum as their only drinking source for one month, following the method previously described [21]. To acclimate the mice, the ethanol concentration was gradually increased by 2% per day over the course of 1 week until reaching 10%. Mice receiving the ethanol solution were assigned to the CAC group (E group): WT (E) and *Adh3^-/-^* (E). Mice provided only water served as controls (C group): WT (C) and *Adh3^-/-^* (C).

### 4.3. Tissue Preparation

After 1 month of ethanol consumption, WT and *Adh3^-/-^* mice were anesthetized, had blood collected from the retro-orbital venousplexus, and were euthanized by cervical dislocation. For light microscopic examination, kidneys were fixed in 10% neutral-buffered formalin (Cat. No. 068-01663, Wako, Osaka, Japan). Within two weeks, the fixed tissues were dehydrated using a vacuum rotary processor (Sakura Finetek Japan Co., Ltd., Tokyo, Japan) and embedded in paraffin. Paraffin blocks were sectioned at a thickness of 2 μm and stained with periodic acid–Schiff (PAS) stain or hematoxylin and eosin (HE) stain for morphological evaluation following the manufacturer’s protocol (PAS stain: periodic acid, Cat. No. 86171; Schiff regent, Cat. No. 40921; sulfurous acid solution, Cat. No. 40941; Mayer’s hematoxylin, Cat. No. 30002. HE stain: Carrazzi’s hematoxylin, Cat. No. 30022; Eosin Y, Cat. No. 31901. Muto Pure Chemicals Co., Ltd., Tokyo, Japan). Digital images were captured using a BZ-9000 All-in-One Microscope (Keyence Corporation, Osaka, Japan).

For electron microscopic examination, 1 mm^3^ kidney samples were fixed in 2.5% glutaraldehyde (Cat. No. 303-2, Nisshin-EM Co., Ltd., Tokyo, Japan; product by TAAB Laboratories Equipment Ltd., Berks, UK) prepared in 0.1 M phosphate buffer (Cat. No. 80362, Muto Pure Chemicals Co., Ltd., Tokyo, Japan). Tissues were then post-fixed in 1% osmium tetroxide (Cat. No. 300, Nisshin-EM Co. Ltd., Tokyo, Japan; product by Heraeus South Africa (Pty) Ltd., Port Elizabeth, South Africa), dehydrated in a graded ethanol series, and embedded in Oken Epok 812 resin (Cat. No. 99-02-1001, Okenshoji Co., Ltd., Tokyo, Japan). Ultrathin sections (70 nm) were prepared using a Leica Ultracut R ultramicrotome (Leica, Wetzlar, Germany), stained with uranyl acetate and lead citrate, and examined using a JEM-1400 Plus electron microscope (JEOL Ltd., Tokyo, Japan).

### 4.4. Measurement of the Ratio of Bowman’s Capsules with Cuboidal PECs and Flat PECs

The number of Bowman’s capsules lined with cuboidal or flat PECs was counted in each group using a mechanical tally counter. All classifications were conducted through visual inspection using a microscope. PECs exhibiting even slight deviations from a flat morphology were classified as cuboidal. Image analysis was performed on hematoxylin and eosin (HE)-stained sections using Adobe Photoshop (version 21.0.1; Adobe Systems, Inc., San Jose, CA, USA).

### 4.5. Measurement of Biochemical Parameters in Serum and Urine

Blood samples were collected and centrifuged at 3000× *g* for 15 min to obtain serum. A mouse metabolic gauge (Shinano Manufacturing Co., Ltd., Tokyo, Japan) was used to collect 24-h urine samples one month after the initiation of ethanol administration. Urine collection was conducted in two intervals: from 10:00 am to 18:00 pm and from 18:00 pm to 12 noon the following day. The concentrations of serum and urinary testosterone were measured using a Testosterone Enzyme Immunoassay Kit (Cat. No. K032-H1, Funakoshi Co., Ltd., Tokyo, Japan; product by Arbor Assays, Ann Arbor, MI, USA). Absorbance was measured with a SpectraMax^TM^ i3x microplate reader (Molecular Devices Japan, Tokyo, Japan) according to the manufacturer’s instructions.

### 4.6. Statistical Analysis

All continuous variables are presented as the mean ± standard deviation (SD). Differences between the control and ethanol-treated groups were analyzed using one-way analysis of variance, followed by Tukey’s Honest Significant Difference test for post hoc comparisons. Statistically significant differences are indicated in each figure. All statistical analyses were performed using JMP software, version 14 (SAS Institute Inc., Cary, NC, USA).

## Data Availability

The original contributions presented in this study are included in the article. Further inquiries can be directed to the corresponding author(s).

## Abbreviations

The following abbreviations are used in this manuscript:

ADH	alcohol dehydrogenase
*Adh3^-/-^*	class III Alcohol dehydrogenase deficient mouse
wild	C57BL/6N mouse
CYP2E1	cytochrome P450 2E1
MEOS	microsomal ethanol oxidizing system
NADH	nicotinamide adenine dinucleotide
ROS	reactive oxygen species
ALD	alcoholic liver disease
PECs	parietal epithelial cells
PPAR γ	peroxisome proliferator-activated receptor γ
GSNO	S-nitrosoglutathione
GSNOR	S-nitrosoglutathione reductase
BUN	urea nitrogen
PAS	periodate acid–Schiff
